# Planetary Health and Infectious Diseases

**DOI:** 10.1093/ofid/ofaf500

**Published:** 2025-11-14

**Authors:** Amy Yomiko Vittor

**Affiliations:** University of Florida, Department of Medicine and Emerging Pathogens Institute, Gainesville, Florida, USA

**Keywords:** emerging pathogens, infectious diseases, medical education, Planetary Health

We are living in an era of profound ecological disruption, marked by climate change, biodiversity loss, and the accumulation of novel compounds such as per- and polyfluoroalkyl substances (PFAS) and microplastics. As physicians and scientists working in Infectious Diseases, we are learning about their health effects and witnessing firsthand how these forces are reshaping our field—altering the geographic distribution of diseases, shifting seasonal transmission patterns, and influencing host-pathogen dynamics.

In response to these challenges, the concept of Planetary Health has emerged as a framework for understanding the interconnectedness of human health and Earth systems. Launched in 2015 with the publication of the Rockefeller Foundation-Lancet Commission on Planetary Health [1], this framework emphasizes that human well-being is inextricably linked to the health of the planet. Despite growing awareness, however, actionable pathways for clinicians and health systems to implement Planetary Health principles remain underdeveloped. While general guidelines and educational initiatives are expanding, specialty-specific blueprints are needed to translate theory into practice.

This special edition on Planetary Health and Infectious Diseases brings together diverse contributions that reflect the breadth of this challenge. Articles examine foundational questions about how to operationalize Planetary Health and One Health, analyze the influence of climate change and pollution on disease emergence, share educational resources, and present actionable interventions.

In a pair of thought-provoking articles, Giraudoux et al [2] and Chemberlin et al [3] challenge readers to view the connection between ecology and health through different ontological lenses. Giraudoux and colleagues explore the tensions inherent in the quadripartite One Health definition (World Health Organization, Food and Agriculture Organization, World Organization for Animal Health, and the United Nations Environmental Program), while Chemberlin and colleagues examine colonialism as a cause of current ecological crises and the contributions that a relational approach can offer to infectious disease research.

Jara et al [4] and Koch et al [5] then analyze the associations between meteorological factors and *Candidozyma auris*—formerly known as *Candida auris*—and nontuberculous mycobacteria, respectively. The literature on the linkages between climate and these important pathogens is relatively scarce. As the clinical burden due to these pathogens increases [6, 7], understanding the underlying drivers is critical for forecasting. In the case of *C. auris*, its rapid, simultaneous appearance in disparate geographic regions has led to the hypothesis that climate change played a role in its emergence through selection for thermotolerant species [8]. In this supplement, Jara et al analyzed more than 800 *C. auris* genomes from symptomatic individuals in New York and New Jersey over 8 years, correlating genetic markers with meteorological variables and suggesting that antifungal resistance genes are associated with high average and minimum temperatures. Meanwhile, Honda et al provide evidence that the distribution of nontuberculous mycobacteria pathogenic to humans is also driven by climate, and forecast their distribution in Hawaii.

In addition to ecological drivers of disease, contributors examine how healthcare itself contributes to greenhouse gas emissions and waste. The production, use, and disposal of antimicrobials, for example, contribute to environmental pollution and climate change. Articles by Hoskins et al [9] and Hojat et al [10] introduce tools to quantify the carbon footprint of hospital-based antibiotic use and to quantify antibiotic community pharmacy disposal, offering pathways to reduce waste and emissions. Whitman et al [11] propose a practical solution to reducing greenhouse gases while promoting health by providing a structured guide to help Infectious Disease physicians counsel their patients on adopting plant-based diets. Miller [12] brings the issue of emerging infectious disease threats on the blood supply to our attention, arguing for a dedicated infectious diseases consultation services within transfusion medicine to better anticipate and manage emerging threats tied to shifting pathogen distributions.

Education is another essential pillar in the advancement of Planetary Health. Formal training in climate and health equips healthcare professionals with the knowledge and skills needed to address the complex interplay between environmental factors and human health. In this supplement, Rosenberg et al [13] propose a model curriculum that can be readily adapted to various domains within Infectious Diseases. Judson et al [14] complement this with an open-access, case-based online curriculum that introduces trainees to key topics including extreme heat, emerging zoonoses, displacement and refugee health, and toxic exposures.

Ultimately, integrating Planetary Health into the practice of Infectious Diseases requires a coordinated, multifaceted approach that bridges research, education, and clinical care. We hope that the articles in this supplement catalyze Planetary Health conversations in Infectious Diseases and aid in developing practical guidelines and solutions that can be incorporated into clinical care. OFID is committed to continuing this conversation, welcoming manuscripts on an ongoing basis that explore the impact of ecological factors on the risk, determinants, distribution, and severity of infectious diseases, how these topics are taught in medical education, and their potential solutions.
